# Supplementary material for: Content-rich biological network constructed by mining PubMed abstracts
Source: BMC Bioinformatics. 2004 Oct 8;5:147. doi: 10.1186/1471-2105-5-147 (PMC528731; doi:10.1186/1471-2105-5-147)
Supplement: Additional File 2 — The original results of the above study (non-essential files are deleted to keep the file size under the limit set by BMC bioinformatics). [file 1471-2105-5-147-S2.bz2 › chilibotAdditionalFile2/dip40/223ID7831290E739/html/SYK_LYN.html]

 


 **SYK** and **LYN** 
  
Found 264 abstracts in PubMed, retrieved 40.  
 

 What does Google say? 
 PDF only 
| .edu only 

---

**Interactive relationship** (e.g. stimulation, inhibition, etc)

**Neutral relationship**- Our data indicate that ligation of Fcgamma receptors activates  **Lyn**  followed by  **Syk**  kinase and in the result multimolecular complex of the kinases and several accompanying tyrosine phosphorylated proteins with Fcgamma receptors is organized leading to local reorganization of actin based skeleton and particle uptake.  Ref: 12056610 Folia Histochem Cytobiol, 2002
- The hantavirus ITAM coprecipitated  **Lyn** ,  **Syk** , and ZAP 70 kinases from T or B cells, while mutagenesis of the ITAM abolished these interactions.  Ref: 12502882 J Virol, 2003
- We find that  **Lyn**  phosphorylates only the first tyrosine whereas  **Syk**  phosphorylates both tyrosines of the BCR immunoreceptor tyrosine based activation motif ITAM.  Ref: 12453414 Mol Cell, 2002
- These LMP2A functions require interactions with the protein tyrosine kinases  **Syk**  and  **Lyn** .  Ref: 11878875 Virology, 2001
- We present a detailed mathematical model of the phosphorylation and dephosphorylation events that occur upon ligand induced receptor aggregation, for a transfectant expressing Fc varepsilon RI,  **Lyn** ,  **Syk**  and endogenous phosphatases that dephosphorylate exposed phosphotyrosines on Fc varepsilon RI and  **Syk** .  Ref: 12217386 Mol Immunol, 2002
- Methyl dihydroxycinnamate DHC, a PTK inhibitor, was more selective for  **Syk**  than  **Lyn** , while staurosporine ST, a broad specificity protein kinase inhibitor, inhibited  **Lyn**  more than  **Syk** .  Ref: 12739161 Pflugers Arch, 2003

**Non-interactive relationship** (e.g. studied together, co-existance, homology, etc.)

- In B CLL cells, BCR cross linking neither induced activation nor enhanced the activities of  **Lyn** ,  **Syk** , p21ras, JNK, p38 MAPK, or Akt kinases.  Ref: 12095152 Int J Hematol, 2002
- We demonstrate that in addition to tyrosine kinases  **Lyn**  and Btk, FcalphaR cross linking also recruits B lymphocyte kinase Blk and spleen tyrosine kinase  [ **SYK** ]   **Syk**  to rafts.  Ref: 12023895 Biochem J, 2002
- Of particular interest is WHI P131, which inhibits JAK3 but not JAK1, JAK2,  **SYK** , BTK,  **LYN** , or IRK at concentrations as high as 350 microM.  Ref: 12188892 Curr Cancer Drug Targets, 2001
- The activity of the kinases that phosphorylate the Fc receptor gamma chain, Fyn and  **Lyn** , as well as the tyrosine kinase  **Syk**  and phosphoinositide 3 kinase was also inhibited by quercetin in a concentration dependent manner, both in whole cells and in isolation.  Ref: 12871380 J Thromb Haemost, 2003
- We have reported previously that deoxygenation of sickle cells stimulates KCC and activates  **Syk**  a  **Syk**  family PTK, but not  **Lyn**  an Src family PTK.  Ref: 12739161 Pflugers Arch, 2003
- In this study, we demonstrate a sequential involvement of  **Lyn**  and  **Syk**  in the phagocytosis of IgG coated particles.  Ref: 12471110 J Immunol, 2002
- Here, we report that the calcium ionophore ionomycin induces a massive Ca2 dependent apoptosis in wildtype DT 40 chicken B lymphoma cells, as well as in BTK deficient, PLCgamma2 deficient and IP3 receptor deficient DT 40 cells, but not in  **LYN**  or  **SYK**  deficient DT 40 cells.  Ref: 12533043 Leuk Lymphoma, 2002
- It is concluded that EGCG inhibits platelet activation, by hindering the thrombin proteolytic activity, and by reducing the agonist induced Ca 2 c increase through inhibition of  **Syk**  and  **Lyn**  activities.  Ref: 12719785 Thromb Haemost, 2003
- Immobilized ophioluxin was used to isolate the glycoprotein VI Fcgamma complex from resting platelets, which also contained Fyn,  **Lyn** ,  **Syk** , LAT, and SLP76.  Ref: 12130642 J Biol Chem, 2002
- Taken together, our data suggests that both  **LYN**  and  **SYK** , but not BTK, FYN or LCK, are crucial mediators of BCR independent Ca2 induced apoptosis in DT 40 lymphoma B cells.  Ref: 12533043 Leuk Lymphoma, 2002
- We investigated the roles of the Src kinase  **Lyn** , the immunoreceptor tyrosine based activation motifs ITAMs on the beta and gamma subunits of Fc epsilon RI, and  **Syk**  itself in the activation of  **Syk** .  Ref: 12646643 J Immunol, 2003
- Stable expression of this  **Lyn**  PLCgamma2 was not accompanied by an increase in substrate hydrolysis in resting cells which followed stimulation and specifically required presence and or activation of  **Syk** , Btk, PI 3K but not BLNK, as established using deficient cell lines or specific inhibitors.  Ref: 12780340 Biochem J< MedlineTA>Biochem J, 2003
- **SYK**  and  **LYN**  mediate B cell receptor independent calcium induced apoptosis in DT 40 lymphoma B cells.  Ref: 12533043 Leuk Lymphoma, 2002
- In contrast, the tumor cells in all cases of lymphocyte predominance Hodgkin rad s disease were positive for Fyn,  **Syk** , BLNK and PLC gamma2, and  **Lyn**  immunostaining was seen in a minority of biopsies.  Ref: 12881301 BloodBlood, 2002
- Under our conditions, hydrogen peroxide induced PI3K and Akt activation was independent of  **Lyn** ,  **Syk** , Cbl, BCAP, or Ras when each was eliminated individually either by mutation or by a specific inhibitor.  Ref: 12627965 Biochemistry, 2003
- Imnunofluorescent studies confirm spatial and temporal distribution of  **Lyn**  and  **Syk**  kinases at different stages of phagocytosis.  Ref: 12056610 Folia Histochem Cytobiol, 2002
- With this powerful approach of reverse genetics, we studied the interaction of the protein tyrosine kinases  **Syk**  and  **Lyn**  with the B cell antigen receptor BCR.  Ref: 12453414 Mol Cell, 2002
- An in vitro binding study revealed that phosphorylation of Tyr 446 by  **Syk**  was likely to create a binding site for the  **Lyn**  SH2 domain in RBL 2H3 cells.  Ref: 12709437 J Biol Chem< MedlineTA>J Biol Chem, 2002
- Reconstitution of  **SYK**  deficient cells with wild type  **SYK**  restored the apoptotic response of the cells to ionomycin, but the expression of FYN or LCK in  **LYN**  deficient cells did not restore the apoptotic response of  **LYN**  deficient cells.  Ref: 12533043 Leuk Lymphoma, 2002
- Activation of MCs through FcepsilonRI is associated with increased expression of activation linked membrane antigens as well as with signaling events involving  **Lyn**  and  **Syk**  kinases, the phosphatidylinositol 3 kinase pathway, Ras pathway, and the phospholipase C protein kinase C pathway.  Ref: 12041664 Int J Hematol, 2002
- In addition, interpreted in the context of the model, experimentally observed differences between the beta and gamma chains with respect to levels of phosphorylation and rates of dephosphorylation indicate that most cellular  **Syk** , but only a small fraction of  **Lyn** , is available to interact with receptors.  Ref: 12646643 J Immunol, 2003
- Downstream of Fc varepsilon RI and its coupled tyrosine kinases,  **Lyn**  and  **Syk** , scores of different proteins and lipids are implicated in these signaling cascades and new players are being identified continuously.  Ref: 12217393 Mol Immunol, 2002
- Sequential translocation of tyrosine kinases  **Lyn**  and  **Syk**  to the activated Fcgamma receptors during phagocytosis.  Ref: 12056610 Folia Histochem Cytobiol, 2002
- In line with this, silver had no or only marginal effect on the tyrosine phosphorylation of FcepsilonRIbeta,  **Lyn** ,  **Syk** , and linker for activation of T cells, the early and crucial events in FcepsilonRI signaling.  Ref: 12244196 J Immunol, 2002
- FcepsilonRI signals by activating proximal non receptor type of protein tyrosine kinases,  **Lyn** ,  **Syk** , Btk and Fyn.  Ref: 12558077 Curr Mol Med, 2003
- Fc epsilon RI activation of mast cells is thought to involve  **Lyn**  and  **Syk**  kinases proximal to the receptor and the signaling complex organized by the linker for activation of T cells LAT.  Ref: 12089510 Nat Immunol, 2002
- Our approach was to build a detailed mathematical model of reactions involving Fc epsilon RI,  **Lyn** ,  **Syk** , and a bivalent ligand that aggregates Fc epsilon RI.  Ref: 12646643 J Immunol, 2003
- **Lyn**  and  **Syk**  kinases are sequentially engaged in phagocytosis mediated by Fc gamma R.  Ref: 12471110 J Immunol, 2002
- Activation of nontransmembrane protein tyrosine kinases, such as  **Lyn**  and  **Syk** , has been shown to be the earliest detectable signaling response to Fc receptor Fc epsilon RI cross linking on mast cells leading to mast cell degranulation.  Ref: 12044809 Eur J Pharmacol, 2002
- BCR proximal signaling occurs within cholesterol and sphingolipid rich plasma membrane microdomains termed lipid rafts, and involves tyrosine kinases such as  **Lyn** ,  **Syk**  and Btk and the adapter molecule SLP65 BLNK.  Ref: 11913944 Int Rev Immunol, 2001
- This phosphorylation was intact in Btk and Cbl deficient DT40 cells, but was drastically suppressed in  **Lyn** ,  **Syk** , or BCAP deficient DT40 cells.  Ref: 12627965 Biochemistry, 2003
- A great deal of research has focused on the earliest steps in this signaling cascade resulting in models which include the participation of  **lyn** ,  **syk** , phospholipase C, protein kinase C and intracellular calcium mobilization.  Ref: 12217403 Mol Immunol, 2002
- Primary signaling domains, also called osmiophilic patches, are recognized by their dark staining with osmium, adjacency to coated pits previously mapped to planar membrane between lamellae and by the characteristic presence of receptor,  **Syk**  and PLCgamma2, but not  **Lyn** .  Ref: 12217393 Mol Immunol, 2002
- Thus,  **Syk**  and  **Lyn**  combine to determine the pathway through which the BCR is coupled to Ca 2 mobilization as well as the magnitude and duration of the Ca 2 flux.  Ref: 12077122 J Biol Chem, 2002
- However, exaggerated positive signaling is also apparent as evidenced by the constitutive phosphorylation of  **Syk**  and phospholipase Cgamma2 in resting  **Lyn**  up up B cells.  Ref: 12486102 J Exp Med, 2002
- Both tyrosine kinases  **Syk**  and  **Lyn** , immuno precipitated from stimulated platelets, are greatly inhibited upon cellular pre incubation with EGCG, which also inhibits the in vitro auto phosphorylation and exogenous activity of these two enzymes purified from rat spleen.  Ref: 12719785 Thromb Haemost, 2003
